# C-3 Substituted Lawsonemonoximates of Holmium(III): Synthesis, Characterization, and Antimicrobial Activity

**DOI:** 10.1155/MBD.2000.147

**Published:** 2000

**Authors:** S. B. Jagtap, S. G. Joshi, G. M. Litake, V. S. Ghole, B. A. Kulkarni

**Affiliations:** 1 Department of Chemistry University of Pune Pune 411 007 India; 2 Department of Pathology & Microbiology D. S. H. Medical College Pune 411004 India

## Abstract

A series of five new metal complexes of Ho(III) with C-3 substituted derivatives of lawsonemonexime (2-hydroxy-1,4-naphthalenediene-1-oxime) were synthesized. The compounds were characterized by melting point, elemental analysis, IR spectroscopy and magnetic susceptibility. The antimicrobial activity of the compounds were determined by disk diffusion method and broth micro-dilution techniques using Mueller Hinton medium against the following organisms: *S. aureus* ATCC 6538P, *Klebsiella pneumoniae*, NCTC 418, *Pseudomonas aeruginosa* ATCC 27833, *Salmonella typhimurium* ATCC 23564, *E. coli* U 1777, *E. coli* HB101, *Proteus morganii* NCIM 2860, *Providencia stuartii* NCIM 2799 and *Acinetobacter baumannii* U 24. The chelates of Ho(III) with lawsonemonoxime and Ho(III) with 3-bromolawsonemonoxime showed a variable antimicrobial activity against all organisms tested except *Pseudomonas* and *Klebsiella* spp. *S. aureus* was found more sensitive to all ligands and chelates tested; but the MIC values of chelates were considerably less; thus having more antimicrobial effect.


### C- 3 SUBSTITUTED LAWSONEMONOXIMATES OF HOLMIUM(III): SYNTHESIS, CHARACTERIZATION, AND ANTIMICROBIAL ACTIVITY

S. B. Jagtap[1], S. G. Joshi[2], G. M. Litake[1], V. S. Ghole*[1], and B. A. Kulkarni[1]

[1] Department of Chemistry, University of Pune, Pune - 411 007. India.
[1] Department of Pathology & Microbiology, D. S. H. Medical College, Pune-411004.
Fax: + 91 20 5651728 <vsghole@chem.unipune.ernet.in>

### ABSTRACT

A series of five new metal complexes of Ho(III) with C-3 substituted derivatives of lawsonemonoxime (2-hydroxy-1,4-naphthalenedione-1-oxime) were synthesized. The compounds were characterized by melting point, elemental analysis, IR spectroscopy and magnetic susceptibility. The antimicrobial activity of the compounds were determined by disk diffusion method and broth micro-dilution techniques using Mueller Hinton medium against the following organisms: *S. aureus* ATCC 6538P, *Klebsiella pneumoniae*, NCTC 418, *Pseudomonas aeruginosa* ATCC 27833, *Salmonella typhimurium* ATCC 23564, *E. coli* U 1777, *E. coli* HB101, *Proteus morganii* NCIM 2860, *Providencia stuartii* NCIM 2799 and *Acinetobacter baumannii* U 24. The chelates of Ho(III) with lawsonemonoxime and Ho(III) with 3-bromolawsonemonoxime showed a variable antimicrobial activity against all organisms tested except *Pseudomonas* and *Klebsiella* spp. *S. aureus* was found more sensitive to all ligands and chelates tested; but the MIC values of chelates were considerably less; thus having more antimicrobial effect.

### INTRODUCTION

Antimicrobial or antineoplastic activity of lawsone was studied by Lime et al [1]. Tripathi et al [2] showed that lawsone exhibits fungicidal activity. Anderson et al [3] studied phthiocol which is the quinone compound associated with certain bacteria. Some oxime derivatives of lawsone and their chelates showed antimicrobial activity. Mhaske et al [4] have reported the antimicrobial activities of 3-nitrolawsonemonoxime, 3-aminolawsonemonoxime and their bivalent metal chelates where as Dandawate [5] and Gaikwad [6] studied the chelates of some rare earths with lawsonemonoxime derivatives. Joshi et al [7] have reported on rare earth studies where as Shen et al [8] studied lanthanide metal complexes for antimicrobial activities. But the chelates of Ho(III) with lawsonemonoximes (figure 1) and their antimicrobial activities are being reported for the first time in the present studies.

A  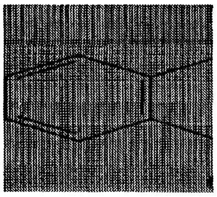 B 

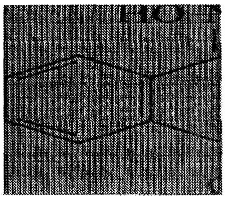

| R | Ligand |
|---|---|
| H | Lawsonemonoxime (HL1) |
| $CH_3$ | Phthiocolmonoxime (HL2) |
| Cl | 3-Chlorolawsonemonoxime (HL3) |
| Br | 3-Bromolawsonemonoxime (HL4) |
| I | 3-Iodolawsonemonoxime (HL5) |

Figure 1: (A) Structure of lawsonemonoxime derivatives (B): General structure of the ligating system

### MATERIALS AND METHODS

### Preparation of the ligands

All the chemicals and solvents used were of analytical grade. Lawsone (2-hydroxy-1,4-naphthalenedione), dichlone (2,3-dichloro-1,4-naphthalenedione) and menadione (2-methyl-1,4-naphthalenedione) were purchased from Fluka (Germany). Phthiocol was synthesized from menadione by Fieser's method [9]. The 3-chlorolawsone was prepared from dichlone. All the ligands (figure 1) used (lawsonemonoxime derivatives) were synthesized by treating the solution of a lawsone derivative with solution of hydroxylamine hydrochloride. The entire mixture was heated at 60°C, cooled and then neutralized with hydrochloric acid (2M) causing precipitation of the corresponding lawsone-1-oxime derivative. The melting points were recorded after recrystallization of the ligands from methanol. The compounds were tested for solubility in water, methanol, DMF, DMSO and acetonitrile.

### Preparation of the Chelates

To a hot solution of 3.0 mmol of ligand (table 1), (0.568 g of lawsonemonoxime, 0.60 g of phthiocolmonoxime, 0.671 g of 3-chlorolawsonemonoxime, 0.804 g of 3-bromolawsonemonoxime and 0.945 g of 3-iodolawsonemonoxime) in 25 mL of ethanol, an aqueous solution of 1.00 mmol of metal(III)chloride hexahydrate (0.379 g of $HoCl_3.6H_2O$) was added. The pH of the mixture was adjusted to 5.5 to 6.5 using aqueous ammonia (1:20 v/v). The mixture was refluxed for 3 h and then cooled overnight. The precipitate was filtered off, washed with water followed by hot methanol and dried in vacuum over fused CaCl2 at ambient temperature. A solubility of the complexes was tested in methanol, water, DMF, DMSO and acetonitrile.

The carbon, hydrogen analysis and residue ($Ho_2O_3$) were determined using a Hosli- Holland C, H Analyzer. The magnetic studies were carried out at room temperature by the Faraday technique using mercury(II) tetrathiocyanatocobaltate as calibrant. The infrared spectra were recorded in nujol mulls on a Perkin- Elmer FTIR Spectrophotometer (Model 1600, range 4000- 450 $cm^{-1}$).

**Table 1.** Elemental analysis and some physical properties of the ligands having antimicrobial activity.

| Ligand | Colour | M.p. | Yield | Elemental Analysis (calc.) found | | |
|---|---|---|---|---|---|---|
| | | (° C) | (%) | % of C | % of H | % of N |
| HL1 | Yellow | 180 | 85 | 63.17 (65.49) | 3.72 (3.70) | 7.45 (7.41) |
| HL2 | Pale yellow | 200 | 95 | 65.35 (65.02) | 4.41 (4.43) | 6.93 (6.91) |
| HL3 | Glossy greenish yellow | 188 | 94 | 53.46 (53.69) | 2.66 (2.68) | 6.23 (6.26) |
| HL4 | Pale greenish yellow | 192 | 90 | 43.49 (44.77) | 2.20 (2.24) | 5.05 (5.22) |
| HL5 | Pale greenish yellow | 158 | 88 | 37.98 (38.10) | 2.10 (1.90) | 4.42 (4.44) |

M.p., melting point, HL1, ($C_{10}H_7O_3N$); HL2, ($C_{11}H_9O_3N$); HL3, ($C_{10}H_6O_3N.Cl$); HL4, ($C_{10}H_6O_3N.Br$); HL5, ($C_{10}H_6O_3N.I$).

**Antimicrobial activity of the compounds**

The antimicrobial activity was determined by the disk diffusion method[10] against the following organisms: *Staphylococcus aureus* ATCC 6538P, *Klebsiella pneumoniae* NCTC 418, *Pseudomonas aeruginosa* ATCC 27833, *Salmonella typhimurium* ATCC 23564, *E. coli* U1777, *E. coli* HB101, *Proteus morganii* NCIM 2860, *Providencia sturtii* NCIM 2799, and *Acinetobacter baumannii* U24.

In brief, Mueller Hinton agar (HiMedia Lab, India) was prepared as per instructions of the manufacturer and plates were poured around 56°C to set and then placed in the incubator at 37°C for overnight to test the sterility of medium. Meanwhile, an overnight nutrient broth culture of each strain was taken and diluted to reach O.D. of 0.5 MacFarland standard with nutrient broth, of which 50 μL was inoculated on plate and spreaded with glass spreader. After 1 h the disks (prepared from Whatman No 42, 6 mm diameter) impregnated with the compounds (prepared in DMSO to water 3:1) were applied and the plates incubated at 37°C for 48 h. The zones of inhibition of growth around disks were measured at the intervals of 18, 24 and 48 h. The method was applied to all strains selected and repeated twice.

**Determination of MICs**

The minimum inhibitory concentrations (MICs) were determined using broth microdilution technique. Serial two fold dilutions of compounds ranging the concentrations from 1024 μg through 32 μg/ mL were prepared in nutrient broth. The inoculum of 50 μL of nutrient broth culture, having O.D. of 0.5 MacFarland standard was inoculated into sterile bumper test tubes (2.5 cm x 15 cm) containing 5 mL of Mueller Hinton broth containing above concentrations of compounds. The tubes were incubated at 37°C for 48 h and observed for any visible growth at the intervals of 18, 24 and 48 h. The MIC was defined as the lowest concentration of compound in the medium, showing complete inhibition of visible growth.

### RESULTS AND DISCUSSION

The interaction of the ligands (table 1) with metal salts in 1:3 molar ratio in ethanol yielded stable solid complexes corresponding to the molecular composition supported by elemental analysis is $HoL_3(H_2O)_2$ (where L = anion of the corresponding ligands). All the complexes (table 2) are soluble in donor solvents such as DMSO and DMF, moderately soluble in solvents like methanol and acetonitrile and insoluble in inert solvents like n-hexane, benzene,1,4-dioxane etc. All the ligands were soluble in methanol, water, DMF, DMSO and acetonitrile.

**Table 2**. Elemental analysis and physical properties of metal chelates exhibiting some antimicrobial activity.

| Compd | Colour | Decom. Temp. | Yield | Elemental Analysis found (Calc.) | | | | μeff. (BM) |
|---|---|---|---|---|---|---|---|---|
| | | (o C) | (%) | % C | % H | % N | % M | |
| Hol | Yellow | 290 | 66.68 | 46.79 (47.07) | 3.00 (2.90) | 5.36 (5.49) | 21.38 (21.55) | 10.08 |
| Ho2 | Orange | 236 | 67.47 | 48.77 (49.08) | 3.12 (3.49) | 5.13 (5.30) | 20.36 (20.42) | 10.30 |
| Ho3 | Turmeric yellow | 296 | 68.54 | 41.01 (41.48) | 2.52 (2.20) | 4.81 (4.84) | 18.79 (18.98) | 10.23 |
| Ho4 | Turmeric yellow | 290 | 69.37 | 36.02 (35.96) | 2.25 (1.99) | 3.98 (4.19) | 16.23 (16.46) | 10.27 |
| Ho5 | Turmeric yellow | 268 | 67.45 | 31.79 (31.52) | 2.01 (1.68) | 3.90 (3.68) | 14.20 (14.43) | 10.12 |

μeff. is magnetic moment (BM). Hol, Ho(III).$(C_10H_6O_3N)_3.2H_2O$; Ho2, Ho(III) $(C_{11}H_8O_3N)_3.2H_2O$; Ho3, Ho(III) $(C_{10}H_5O_3NCl)_3.2H_2O$; Ho4, Ho(III) $(C_{10}H_5O_3N.Br)_3.2H_2O$; Ho5, Ho(III) $(C_{10}H_5O_3N.I)_3.2H_2O$.

Selected IR bands of the ligands and complexes are shown in table 3. Ligands exhibit a medium broad band at 3100-3600 $cm^{-1}$ which is due to the (O-H) vibration of oximino and phenolic hydroxyls. On complexation, this band is further broadened due to overlap of the (O-H) stretching frequency of coordinated molecules[5]. The (C=O) stretching frequency at 1600-1630 $cm^{-1}$ found in the IR spectra of ligands is shifted to a lower frequency region by 20-30 $cm^{-1}$ after complexation. This indicates redistribution of electron density in the quinonoid ring i.e. weakening of bond.

The band at 1570-1590 $cm^{-1}$ in the spectra of ligands is assigned to (C=N) vibrations, it is shifted to lower frequency by 40-70 $cm^{-1}$ in the complexes (table 3), which indicates a coordination through the oximino nitrogen. A quinone absorption is observed at 1285-1295 $cm^{-1}$.

**Table 3.** Selected IR bands of ligands and metal chelates showing antimicrobial activity ($cm^{-1}$).

| Compd | (O-H) | (C=O) | (C=N) | Quinone absorption | (C-O) | (N-O) | (C-X) | (Ho-O) |
|---|---|---|---|---|---|---|---|---|
| HL1 | 3362, 3155 | 1630 | 1576 | 1293 | 1211 | 1050 | – | – |
| Hol | 3288 | 1587 | 1537 | 1287 | 1219 | 1052 | – | 463 |
| HL2 | 3275, 3100 | 1620 | 1587 | 1296 | 1205 | 1052 | – | – |
| Ho2 | 3364 | 1585 | 1526 | 1294 | 1225 | 1057 | – | 464 |
| HL3 | 3412, 3375, 3100 | 1604 | 1577 | 1286 | 1211 | 1050 | 694 | – |
| Ho3 | 3177 | 1581 | 1520 | 1290 | 1224 | 1059 | 691 | 465 |
| HL4 | 3325, 3200, 3100 | 1623 | 1589 | 1287 | 1210 | 1055 | 693 | – |
| Ho4 | 3354 | 1580 | 1519 | 1286 | 1223 | 1060 | 689 | 466 |
| HL5 | 3325, 3187, 3100 | 1620 | 1585 | 1286 | 1224 | 1051 | 692 | – |
| Ho5 | 3386 | 1582 | 1519 | 1285 | 1222 | 1053 | 680 | 472 |

X = halogen

The (C-O) stretching frequency for the ligand is found at 1210 $cm^{-1}$, which is shifted to the higher frequencies by 10-35 $cm^{-1}$ in the complexes, which indicates coordination through the phenolato oxygen. The (N-O) stretching frequency is detected at 1050 $cm^{-1}$ which showed a notable shift to a higher frequncy region by 5-20 $cm^{-1}$ after complexation, confirming the coordination through the oximino nitrogen (Piperpont et al)[11]. Absorption at 688- 695 $cm^{-1}$ is assigned to (C-X); after complexation this band is shifted to lower region by 3-10 $cm^{-1}$.

A band at 460-480 $cm^{-1}$ of medium intensity is observed in the spectra of complexes which is assigned to the (Ho-O) stretching band. All the complexes showed slightly smaller magnetic moments than the calculated value of free Ho (III) ion (10.6 BM) suggesting that 4f electrons in these compounds do not participate in complex formation.

**Antimicrobial activity of the ligands and their metal chelates**

No zone of inhibition of growth was observed in any of the culture plates against any of the compound by the end of 48 h, showing that organisms tolerate and survive the disk concentrations (30 μg). So the concentrations of compounds used for MIC were more than 30

μg/mL. The MIC values of all the compounds tested against various microorganisms is shown in table 4. It is apparent from table 4 that the MIC values are very high for each organism, explaining the extent of resistance offered by them, against these compounds.

**Table 4.** Antimicrobial activity (MIC in μg/mL) of ligands and their metal complexes against various microorganisms.

| Compd | 1 | 2 | 3 | 4 | 5 | 6 | 7 | 8 | 9 |
|---|---|---|---|---|---|---|---|---|---|
| HL1 | <512 | >1024 | 512 | >1024 | >1024 | >1024 | >1024 | <512 | >1024 |
| HL2 | 256 | >1024 | 512 | >1024 | 256 | >1024 | >1024 | >1024 | >1024 |
| HL3 | >1024 | >1024 | >1024 | >1024 | 512 | >1024 | >1024 | 512 | >1024 |
| HL4 | 256 | 256 | >1024 | >1024 | 512 | >1024 | >1024 | 512 | >1024 |
| HL5 | >1024 | >1024 | >1024 | >1024 | 256 | >1024 | >1024 | <512 | >1024 |
| Hol | 256 | 512 | 256 | >1024 | 512 | >1024 | <512 | 128 | >1024 |
| Ho2 | 128 | >1024 | 256 | >1024 | 128 | >1024 | >1024 | >1024 | >1024 |
| Ho3 | >1024 | >1024 | >1024 | >1024 | <128 | >1024 | >1024 | 128 | >1024 |
| Ho4 | 128 | <128 | 512 | >1024 | <128 | >1024 | >1024 | 256 | 128 |
| Ho5 | >1024 | >1024 | >1024 | >1024 | 512 | >1024 | >1024 | <256 | >1024 |

HL, ligand; Ho, metal complex; 1, *Acinetobacter*; 2, *Proteus*; 3, *Salmonella*; 4, *Klebsiella*; 5, *S. aureus*; 6, *Pseudomonas*; 7, *E.coli*; 8, *E. coli* HB101; 9, *Providencia.*

*S. aureus* is fairly sensitive to all compounds except lawsonemonoxime, when compared with other organisms tested. *Acinetobacter* was showing moderate MIC values against lawsonemooxime, phthiocolmonoxime, Ho(III) lawsonemonoximate, Ho(III) bromolawsonemonoximate; while against all other compounds, the MIC was more than 1024 μg/ mL. This may explain an inherited resistance of these organisms for these compounds. The chelates namely, Ho(III) lawsonemonoximate, Ho(III) chlorolawsonemonoximate, Ho(III) bromolawsonemonoximate, and Ho(III) iodolawsonemonoximate shown excellent antimicrobial activity against *E. coli*; wherein the organism was resistant to their corresponding ligands (table 4). *Acinetobacter* had also a moderate antimicrobial activity against metal complexes of the ligands, to whom the organism was earlier resistant. So we presumed that the antimicrobial activity is due to bonding of metal complexes between the atom of metal ion and the enzymes of microbial cell; which must be leading to inhibition of microbial growth. The exact mechanism of activity needs to be studied carefully. But looking at further uses of f block (rare earth) series in biological systems, this study may turn helpful in the near future.

**ACKNOWLEDGEMENT**

This work was supported by a Department of Chemistry, University of Pune, through a University Grants Commission's minor research project. The author thank Department of Chemistry, University of Pune; & Department of Microbiology and Pathology of DSH Medical College & Hospital for the facilities provided to carry out this work.

**REFERENCES**

1. Lime, O.G. de, Coelho, J. S., Albuquerque, I.L de, Mello, J. F. de, Martin, D. G., Lacerda, A. L., Moraes Souza, M. A. de., *Rev. Inst. Antibiot.* (Recife), **11**, 21-26 (1971).
2. Tripathi, R. D., Srivastava, H. S., Dixit, S. N., *Experientia*, **34**, 51-52 (1978).
3. Anderson, R. J., Newman, M. S., *J. Biol. Chem.*, **101**, 773 (1933).
4. Mhaske, R. B., Kulkarni, M., Joshi, S. B., Kshirsagar, V. G., Pradhan, S. P. *Ind. J. Pharm. Sci.*, **61(5)**, 293-296 (1999).
5. Dandawate, F. V., Kodolikar, J. G., Kelkar, V. D., Kulkarni, B. A., *Themochim. Acta*, **241**, 103 (1994).
6. Gaikwad, S. D., *Ph. D. Thesis*, University of Pune (India), (1997).
7. Joshi, C., R., Jagtap, G. S., Chalgeri, S. V.. *Ind. J. Pharm. Sci.*, **49**, 188-190 (1987).
8. Shen Xu, Li , Jiang, H., Xie, Y. *Synth. React. Inorg. Met. Org. Chem.* **25**, 1239-47 (1995)
9. Fieser, L. F., *J. Biol. Chem.*, **133**, 391 (1940).
10. National Committee for Clinical Laboratory Standards, *NCCLS documents*, **M2-A4** (1990).
11. Piperpont, C. G., Downs H. M., Rukavina, T. G., *J. Am. Chem. Soc.* **96**, 5573 (1974).

**Received: May 30, 2000 - Accepted: June 6, 2000 - Received in publishable format: June 9, 2000**

